# Synergistic impact of immuno-nutritional and hypoxia-metabolic disturbances on post-stroke epilepsy: a “Two-Hit” prediction model and web-based risk calculator

**DOI:** 10.3389/fnut.2026.1759899

**Published:** 2026-02-26

**Authors:** Shichao Liu, Risheng Liang

**Affiliations:** 1 Department of Neurosurgery, Fujian Medical University Union Hospital, Fuzhou, Fujian, China; 2 Fujian Institute of Neurosurgery, Fuzhou, China

**Keywords:** clinical prediction model, C-reactive protein to albumin ratio, immuno-nutritional index, lactate to albumin ratio, metabolic disturbance, post-stroke epilepsy

## Abstract

**Background:**

Post-stroke epilepsy (PSE) is a severe complication characterized by significant heterogeneity. Traditional anatomical models often fail to identify patients with high metabolic risk but minor structural injury. Based on the concept that systemic metabolic and nutritional disturbances exacerbate neuronal excitability, we proposed a “Two-Hit” hypothesis: an acute immune-inflammatory hit combined with a hypoxia-metabolic hit acts upon nutritionally compromised brain tissue to drive epileptogenesis. This study aims to evaluate the synergistic value of the Immuno-Nutritional Index (C-reactive protein to Albumin Ratio, CAR) and Hypoxia-Nutritional Index (Lactate to Albumin Ratio, LAR) in predicting PSE.

**Methods:**

We conducted a multi-center retrospective cohort study involving 21,459 acute ischemic stroke patients. CAR and LAR were calculated from admission biomarkers to quantify immuno-nutritional and hypoxia-metabolic status. Restricted cubic splines (RCS) were used to model non-linear dose–response relationships. A “Two-Hit” multivariate prediction model was constructed, and its incremental value over baseline clinical features was assessed using the Net Reclassification Improvement (NRI) and Integrated Discrimination Improvement (IDI). A web-based risk calculator was developed for clinical translation.

**Results:**

During a one-year follow-up, 936 patients (4.36%) developed PSE. CAR exhibited a J-shaped relationship with epilepsy risk, reflecting an inflammatory threshold, while LAR showed a bell-shaped association, indicating a “metabolic hyper-excitatory state”. A significant synergistic effect was observed: patients with concurrent elevations in both indices (“Double High”) had a 13.5% incidence rate compared to 2.4% in the “Double Low” group. The “Two-Hit” model achieved an AUC of 0.888, significantly outperforming single-marker and baseline models (NRI 0.820, *p* < 0.001). Importantly, these nutritional indices maintained predictive value even in patients with minor stroke severity (NIHSS < 4).

**Conclusion:**

The CAR and LAR are potent synergistic predictors of PSE, supporting a “Two-Hit” mechanism involving immuno-metabolic disturbances. The developed web-based calculator serves as a valuable preliminary screening tool to identify metabolically high-risk patients. While the model demonstrates robust internal validity, external validation is warranted before widespread clinical adoption. These findings also suggest that optimizing immuno-nutritional management may act as a novel neuroprotective strategy.

## Introduction

Acute ischemic stroke (AIS) remains a leading cause of long-term disability and mortality globally (1–3). As one of the most common and severe delayed neurological complications following AIS, post-stroke epilepsy (PSE) not only significantly impedes neurological recovery and exacerbates cognitive dysfunction but is also closely associated with increased rates of stroke recurrence and all-cause mortality (4–6). Although previous studies have established stroke severity (e.g., National Institutes of Health Stroke Scale [NIHSS] scores) and anatomical infarct location (particularly cortical involvement) as potent predictors of PSE, the incidence of PSE manifests significant heterogeneity in clinical practice (7, 8). A subset of patients develops epilepsy despite having non-severe radiographic presentations or lacking typical cortical structural damage. This phenomenon suggests that, beyond macroscopic structural brain injury, systemic pathophysiological alterations in the microenvironment—specifically immune-inflammatory responses and metabolic stress—may play a more insidious yet critical role in the formation and activation of epileptogenic networks. These factors are often underestimated in traditional anatomical prediction models (9).

Recent evidence increasingly indicates that the epileptogenic process following stroke is a complex cascade involving blood–brain barrier (BBB) disruption, neuroinflammatory activation, and cerebral metabolic dysregulation (6, 10, 11). Based on this, we propose a “Two-Hit” hypothesis for the pathogenesis of PSE: an acute “immune-inflammatory hit” combined with a tissue-level “hypoxia-metabolic hit” acts upon susceptible, nutritionally compromised brain tissue, thereby lowering the seizure threshold. Specifically, elevated C-reactive protein (CRP) reflects a systemic inflammatory response capable of directly compromising BBB integrity (12, 13); lactate accumulation signifies tissue hypoxia and anaerobic metabolism, leading to neuronal excitotoxicity (14, 15). Meanwhile, serum albumin serves not only as a critical neuroprotective protein maintaining colloid osmotic pressure and exerting antioxidant and anti-inflammatory effects but also as a sensitive surrogate marker for the body’s nutritional reserve. Under the metabolic stress of acute stroke, pre-existing malnutrition or acute nutritional depletion significantly impairs the reparative capacity of the neurovascular unit (16). Consequently, single biomarkers are often insufficient to comprehensively capture this pathological imbalance between “inflammatory consumption” and “nutritional defense.” Composite indices integrating damage factors with defense factors may therefore possess superior predictive value.

To test this hypothesis, this study introduces two composite biomarkers derived from routine admission blood tests: the CRP to Albumin Ratio (CAR, representing the immuno-nutritional index) and the Lactate to Albumin Ratio (LAR, representing the hypoxia-nutritional index). While CAR has been extensively studied in oncology and sepsis prognosis (17, 18), and LAR has demonstrated value in critical care settings (19–21), no study has yet systematically explored the synergistic effects and non-linear characteristics of these two indices in predicting PSE. This study aims to evaluate, for the first time, the independent and synergistic predictive value of CAR and LAR for secondary epilepsy within 1 year after stroke through a large-scale, multi-center retrospective cohort analysis. We specifically focus on exploring the non-linear dose–response relationships between these biomarkers and epilepsy risk, validating the robustness of the “Two-Hit” mechanism across different clinical subgroups, and constructing a user-friendly web-based risk calculator. This tool aims to provide a novel clinical instrument for identifying “metabolically high-risk” patients who are often overlooked by traditional anatomical assessments.

## Methods

### Study design and data source

**Figure 1:**
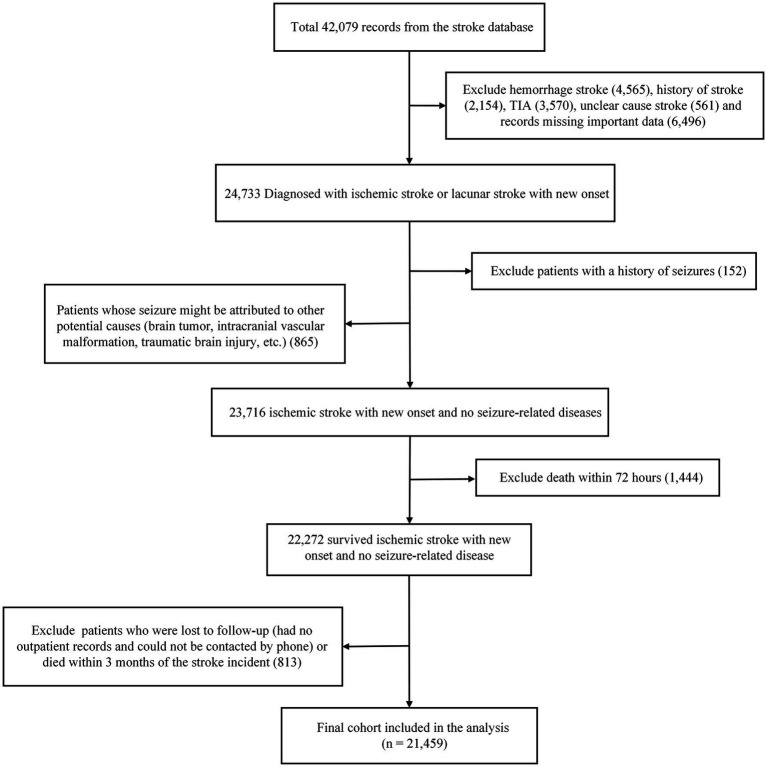
Flowchart of study participant selection.

This study was designed as a retrospective secondary analysis based on a publicly available dataset. The original data were derived from a study published by Liu et al. (8), which aimed to construct a prediction model for secondary epilepsy within 1 year in patients with AIS. According to the original authors’ data availability statement, the complete and de-identified dataset is deposited in the Dryad Digital Repository (22). The dataset aggregates comprehensive clinical data from AIS patients across multiple medical centers in Chongqing, China, spanning from 2013 to 2022. The reliability and quality of the data have been confirmed in subsequent independent validation studies (23). Building upon this high-quality data source, we included a total of 21,459 patients from the original cohort for analysis (screening process shown in Figure 1). During the screening, we strictly adhered to the criteria of the original study: inclusion criteria encompassed a diagnosis of AIS confirmed by head CT or MRI, with complete follow-up records and relevant clinical data; exclusion criteria included a history of epilepsy, hemorrhagic transformation, other non-ischemic brain diseases, and missing key research variables.

Regarding ethical compliance, as this study utilized only publicly available and fully de-identified secondary data, it was exempt from ethical review and patient informed consent requirements in accordance with relevant regulations and the principles of the Declaration of Helsinki. Data acquisition and processing strictly complied with the terms of use and data sharing agreements of the Dryad platform.

### Reporting guidelines and quality assessment

This study is reported in strict accordance with the TRIPOD (Transparent Reporting of a multivariable prediction model for Individual Prognosis or Diagnosis) statement (24). Furthermore, the risk of bias and model applicability were rigorously evaluated using the PROBAST (Prediction model Risk Of Bias ASessment Tool) framework (25). The completed TRIPOD checklist and the detailed PROBAST assessment are provided in Supplementary Tables S1, S2, respectively.

### Data extraction and variable definition

To comprehensively assess patient clinical characteristics, we extracted detailed baseline variables from the original dataset. Demographic characteristics included age and gender; medical history and comorbidities covered hypertension, diabetes mellitus, coronary heart disease, atrial fibrillation, hyperlipidemia, and fatty liver. Regarding stroke clinical characteristics and complications, admission NIHSS scores, cerebral herniation, and hydrocephalus were recorded. Neuroimaging features detailed the anatomical location of the infarct, including the frontal lobe, temporal lobe, parietal lobe, occipital lobe, basal ganglia, and brainstem. To validate anatomical risk, involvement of any region among the frontal, temporal, parietal, or occipital lobes was defined as “cortical involvement.” Laboratory indicators extracted included routine blood tests (white blood cell count, platelet count), biochemical markers (CRP, serum albumin, blood lactate, glycosylated hemoglobin, creatinine), coagulation markers (D-dimer), and electrolytes (sodium, calcium) collected at admission. To quantify the “Two-Hit” effect, we calculated two core composite indices based on raw laboratory metrics: the Immuno-Nutritional Index was defined as the ratio of CRP (mg/L) to serum albumin (g/L) (CAR); the Hypoxia-Nutritional Index was defined as the ratio of blood lactate (mmol/L) to serum albumin (g/L) (LAR). The primary endpoint of this study was defined as secondary epilepsy occurring within 1 year of follow-up.

### Statistical analysis

For baseline characteristics, continuous variables with non-normal distribution were expressed as medians (interquartile range [IQR]) and compared between groups using the Mann–Whitney U test; categorical variables were expressed as frequencies (percentages) and compared using the Chi-square test. Given the narrow numerical ranges and skewed distributions of CAR and LAR, Z-score standardization was applied to these metrics to enhance the interpretability and numerical stability of regression coefficients. Consequently, odds ratios (OR) in all subsequent regression models represent the change in risk per 1-standard deviation (SD) increase in the index.

### Feature selection strategy

To rigorously validate the selection of biomarkers and exclude potential *post hoc* optimization bias, we employed a dual-method feature selection strategy prior to multivariable modeling: (1) Least Absolute Shrinkage and Selection Operator (LASSO) regression was utilized to penalize model complexity and identify non-redundant predictors with non-zero coefficients; and (2) Random Forest (RF) analysis was performed to rank variables based on the Mean Decrease in Gini impurity to assess their predictive importance. All clinical variables showing statistical significance (*p* < 0.05) in the univariate analysis were entered into these screening models.

### Multivariable modeling and validation

We employed multivariable logistic regression models to evaluate the independent associations of CAR and LAR with PSE. The multivariate models were adjusted for age, gender, NIHSS score, cortical involvement, and other potential confounders identified by the feature selection process. To explicitly address potential concerns regarding mathematical coupling arising from the shared denominator (albumin), we calculated the Variance Inflation Factor (VIF) to assess multicollinearity between CAR and LAR. Furthermore, sensitivity analyses were performed by comparing the “Two-Hit” model (utilizing ratio indices) against a “Decomposed Model” utilizing individual biomarkers (CRP, lactate, and albumin modeled as separate covariates) to validate that the ratio formulations captured synergistic information beyond their constituent components.

To explore non-linear relationships, restricted cubic spline (RCS) models were applied to analyze the dose–response relationship between CAR/LAR and epilepsy risk, with four knots specified. Additionally, Spearman correlation analysis was used to assess the correlation between CAR and LAR, and a heatmap of epilepsy incidence was constructed by grouping both indices into quartiles to visually validate the synergistic effect of the “Two-Hit” hypothesis.

To evaluate the incremental predictive value, three multivariate prediction models were constructed: a Baseline Model, a Single-Marker Model, and a Two-Hit Model. Model discrimination was assessed using the area under the receiver operating characteristic curve (AUC), along with the Net Reclassification Improvement (NRI) and Integrated Discrimination Improvement (IDI) to measure improvements in risk stratification. To ensure robust confidence intervals, NRI and IDI calculations utilized 1,000 bootstrap resampling iterations.

### Internal validation and clinical utility

To rigorously assess model performance and correct for potential overfitting, we performed internal validation using the bootstrap method with 1,000 resamples. We calculated the optimism-corrected AUC, calibration slope, and Brier score. Clinical utility was measured via Decision Curve Analysis (DCA) to assess net benefit across different threshold probabilities. Finally, based on the final multivariate logistic regression model, we developed an HTML-based interactive web risk calculator to facilitate clinical translation.

All statistical analyses were performed using Python (version 3.11.5) and R (version 4.4.2), with a two-sided *p*-value of less than 0.05 considered statistically significant.

## Results

### Baseline characteristics of the study population

This study ultimately included 21,459 patients with AIS for analysis. During the one-year follow-up period, 936 patients (4.36%) developed PSE, while the remaining 20,523 patients did not. As shown in Table 1, compared to the non-epilepsy group, patients in the epilepsy group were slightly younger (median age: 66 vs. 67 years, *p* = 0.01) and exhibited more severe neurological deficits at admission (median NIHSS score: 11 vs. 7, *p* < 0.001). Regarding anatomical distribution, the epilepsy group demonstrated significantly higher rates of cortical involvement, specifically in the frontal (15.4% vs. 3.8%), temporal (11.6% vs. 2.6%), and parietal lobes (10.3% vs. 2.8%) (all *p* < 0.001). In terms of immuno-metabolic biomarkers, the epilepsy group presented a “Two-Hit” pattern characterized by concurrent systemic inflammation and metabolic stress. Specifically, white blood cell count, CRP, and lactate levels were significantly elevated, while albumin levels were significantly reduced in the epilepsy group (all *p* < 0.05). Consequently, the derived composite indices were markedly higher in epilepsy patients: the median Immuno-Nutritional Index (CAR) was nearly four times that of the non-epilepsy group (0.81 vs. 0.22, *p* < 0.001), and the Hypoxia-Nutritional Index (LAR) also showed a significant upward trend (0.07 vs. 0.06, *p* < 0.001).

**Table 1:** Baseline characteristics of patients stratified by post-stroke epilepsy status.

Variable	Non-Epilepsy*	Epilepsy*	*p*-value
Age of the patients (years)	67.00 (59.00–76.00)	66.00 (56.00–75.00)	0.01
National Institutes of Health Stroke Scale	7.00 (6.00–9.00)	11.00 (10.00–13.00)	<0.001
White blood cell count (×10^9^/L)	8.10 (7.50–8.90)	11.65 (9.30–13.70)	<0.001
Platelet count (×10^9^/L)	187.80 (174.90–203.30)	180.15 (164.75–199.90)	<0.001
Hemoglobin A1c (%)	6.40 (6.00–7.20)	6.70 (6.10–7.40)	<0.001
Creatinine (μmol/L)	75.30 (66.40–86.50)	77.60 (71.20–89.90)	<0.001
Serum Sodium (mmol/L)	138.70 (137.70–139.60)	138.40 (138.00–138.90)	<0.001
Serum Calcium (mmol/L)	2.20 (2.20–2.20)	2.20 (2.20–2.23)	<0.001
D-dimer (ng/mL)	0.89 (0.65–1.42)	1.99 (1.38–7.24)	<0.001
Albumin (g/L)	41.20 (39.60–42.40)	40.90 (38.90–42.60)	0.02
C-reactive protein (mg/L)	8.90 (4.50–17.60)	33.50 (20.88–88.62)	<0.001
Lactate (mmol/L)	2.40 (2.30–2.70)	2.80 (2.60–3.00)	<0.001
CAR	0.22 (0.11–0.44)	0.84 (0.52–2.18)	<0.001
LAR	0.06 (0.06–0.07)	0.07 (0.07–0.07)	<0.001
Gender (male)	10,271 (50.0%)	572 (61.1%)	<0.001
Hypertension	14,097 (68.7%)	654 (69.9%)	0.47
Diabetes	6,993 (34.1%)	329 (35.1%)	0.52
Coronary artery disease	9,328 (45.5%)	344 (36.8%)	<0.001
Atrial fibrillation	1,930 (9.4%)	113 (12.1%)	0.01
Hyperlipidemia	4,319 (21.0%)	150 (16.0%)	<0.001
Fatty liver disease or steatosis	4,104 (20.0%)	141 (15.1%)	<0.001
Large vessel disease	5,271 (25.7%)	234 (25.0%)	0.67
Frontal lobe of the brain	836 (4.1%)	83 (8.9%)	<0.001
Temporal lobe of the brain	571 (2.8%)	65 (6.9%)	<0.001
Parietal lobe of the brain	595 (2.9%)	68 (7.3%)	<0.001
Occipital lobe of the brain	361 (1.8%)	34 (3.6%)	<0.001
Basal ganglia	910 (4.4%)	58 (6.2%)	0.01
Brainstem	256 (1.2%)	16 (1.7%)	0.28
Cerebral herniation	161 (0.8%)	20 (2.1%)	<0.001
Hydrocephalus	223 (1.1%)	58 (6.2%)	<0.001

*Data are median [interquartile range (IQR)] for continuous variables or number (%) for categorical variables.

CAR, C-reactive protein to albumin ratio; LAR, lactate to albumin ratio.

### Data-driven validation of biomarker selection

The selection of CAR and LAR was primarily driven by our specific pathophysiological hypothesis: the “Two-Hit” mechanism involving immuno-nutritional and hypoxia-metabolic disturbances. While other hematological parameters (e.g., leukocyte count) were also elevated in the epilepsy group, we prioritized CRP and lactate ratios due to their specific mechanistic roles in blood–brain barrier disruption and neuronal hyperexcitability, which are central to epileptogenesis.

Crucially, this biological rationale was corroborated by our machine learning feature selection analysis (Supplementary Figure S1). In the LASSO regression, both CAR and LAR retained non-zero coefficients at the optimal lambda value, indicating non-redundancy. In the Random Forest model, CAR ranked as the third most important predictor (Importance Score: 0.155), and LAR ranked fifth (0.108), outperforming established risk factors such as age and D-dimer. The fact that CAR was identified as a top predictor alongside WBC confirms that the composite “Immuno-Nutritional” status—reflecting the balance between systemic inflammation (insult) and nutritional reserve (defense)—offers distinct prognostic information not fully encompassed by standard metrics of infection (WBC) or stroke severity (NIHSS).

### Association of CAR and LAR with PSE

To assess the independent predictive value of the biomarkers, univariate and multivariate logistic regression analyses were conducted (Table 2). Given the relatively narrow ranges of these indices, data were standardized, and reported OR represent the change in risk per 1-SD increase. In unadjusted models, both CAR (OR 2.03, 95% CI 1.95–2.11) and LAR (OR 1.62, 95% CI 1.55–1.70) demonstrated strong correlations with PSE risk. After adjusting for potential confounders including age, gender, NIHSS score, cortical involvement (frontal/temporal/parietal), and electrolyte levels, these two indices retained independent predictive value. Multivariate analysis revealed that for every 1-SD increase in CAR, epilepsy risk increased by 1.96-fold (adjusted OR 1.96, 95% CI 1.86–2.06, *p* < 0.001); for every 1-SD increase in LAR, risk increased by 1.71-fold (adjusted OR 1.71, 95% CI 1.62–1.81, *p* < 0.001). For clinical translation, the full specifications of the parsimonious model used for the web calculator—including the model intercept and regression coefficients—are explicitly provided in Supplementary Table S3.

**Table 2:** Independent association of CAR and LAR with post-stroke epilepsy.

Variable	Unadjusted OR (95% CI)	Unadjusted *P*	Adjusted OR (95% CI)	Adjusted *P*
CAR (Per SD increase)	2.03 (1.95–2.11)	<0.001	1.96 (1.86–2.06)	<0.001
LAR (Per SD increase)	1.62 (1.55–1.70)	<0.001	1.71 (1.62–1.81)	<0.001

OR, odds ratio; CI, confidence interval; SD, standard deviation; CAR, C-reactive protein to albumin ratio; LAR, lactate to albumin ratio.

### Sensitivity analysis of conceptual independence

To rigorously assess the conceptual independence of CAR and LAR and rule out mathematical coupling due to the shared denominator (albumin), we performed specific validation analyses. First, Variance Inflation Factor (VIF) analysis revealed low values for CAR (1.42) and LAR (1.12), well below the threshold of 5, thus ruling out significant multicollinearity. Second, in a comparative sensitivity analysis, the “Two-Hit Ratio Model” (utilizing composite indices, AUC 0.888) demonstrated predictive performance comparable to a “Decomposed Model” incorporating CRP, lactate, and albumin as separate covariates (AUC 0.892). This finding confirms that the composite indices effectively capture the synergistic prognostic information of their components while offering greater clinical interpretability (Supplementary Table S4).

### Non-linear dose–response relationship and correlation

**Figure 2:**
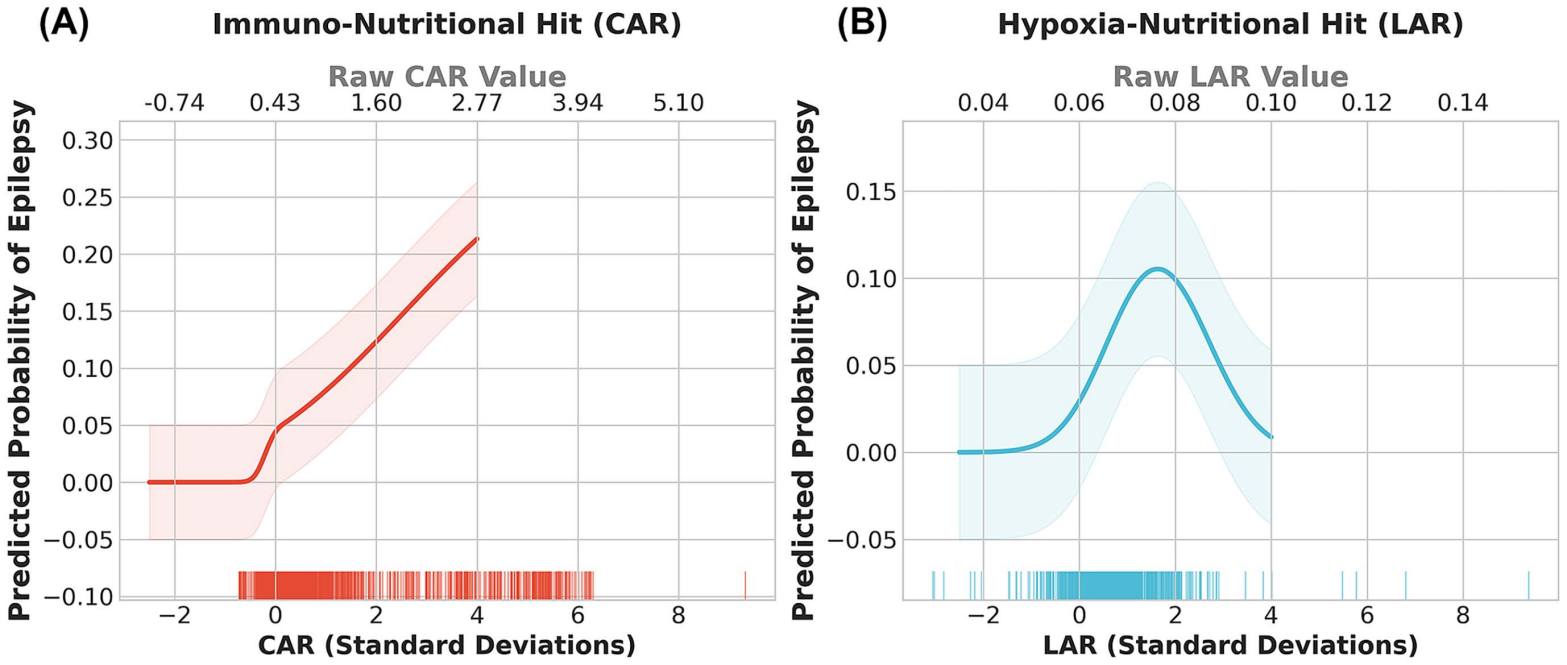
Non-linear dose–response relationships between immuno-nutritional and hypoxia-nutritional indices and the risk of post-stroke epilepsy. Restricted cubic spline models were used to visualize the association between biomarkers and the predicted probability of epilepsy. The solid lines represent the estimated probability, and the shaded areas indicate the 95% confidence intervals. **(A)** The “J-shaped” association between the immuno-nutritional index (CAR) and epilepsy risk, showing a sharp risk increase as CAR exceeds the population mean. **(B)** The “Bell-shaped” (inverted U-shaped) association between the hypoxia-nutritional index (LAR) and epilepsy risk, with the highest risk observed at moderate elevations of LAR. CAR, C-reactive protein to albumin ratio; LAR, lactate to albumin ratio.

**Figure 3:**
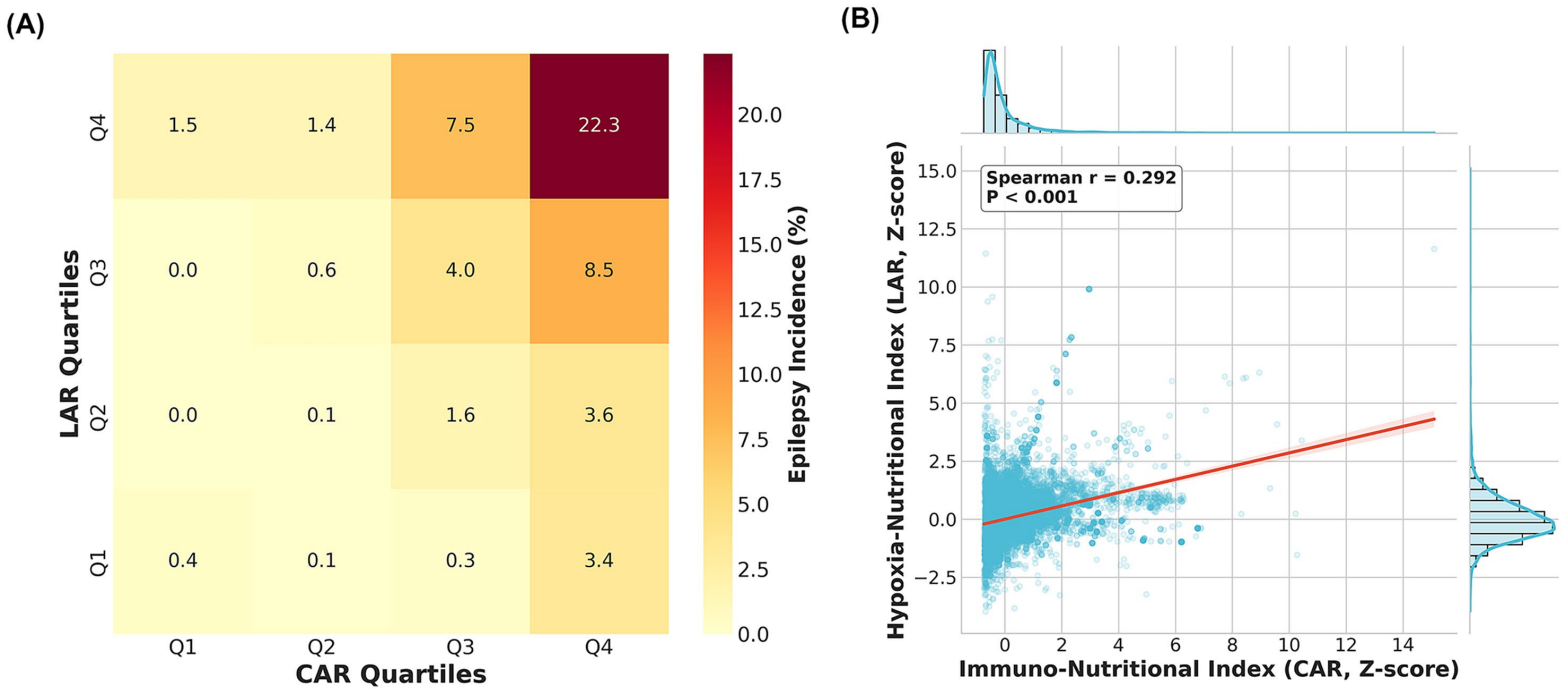
Correlation and synergistic “two-hit” effect of CAR and LAR on post-stroke epilepsy. **(A)** Heatmap illustrating the incidence of epilepsy (%) stratified by quartiles (Q1–Q4) of CAR and LAR. The color intensity moves from light yellow (low risk) to dark red (high risk), revealing a diagonal gradient that supports the synergistic effect of the two indices. The highest incidence (22.3%) is observed in the “double-high” group (Q4–Q4). **(B)** Scatter plot with marginal histograms showing the Spearman correlation analysis. A significant positive linear relationship was observed between standardized CAR and LAR levels (*r* = 0.292, *p* < 0.001). CAR, C-reactive protein to albumin ratio; LAR, lactate to albumin ratio.

Restricted cubic spline models were utilized to visualize the non-linear dose–response relationships between these indices and epilepsy risk (Figure 2). The immuno-nutritional hit (CAR) exhibited a typical “J-shaped” non-linear association (Figure 2A): epilepsy risk remained stable at lower levels but accelerated sharply once the index exceeded the population mean (0 SD), suggesting a threshold effect of systemic inflammation on the epileptogenic process. Notably, the hypoxia-nutritional hit (LAR) displayed a unique “bell-shaped” (inverted U-shaped) relationship (Figure 2B): risk peaked at moderate elevations (approximately 0.10 SD) but paradoxically declined at extremely high values (> 4 SD). This phenomenon suggests complex biological mechanisms, potentially related to seizure suppression by severe metabolic acidosis or competing risks such as mortality. Furthermore, Spearman correlation analysis (Figure 3A) showed a significant positive correlation between standardized CAR and LAR (*r* = 0.385, *p* < 0.001), indicating that while the two are interrelated, they represent distinct pathophysiological pathways, supporting their joint inclusion in prediction models.

### Synergistic effect of the two-hit mechanism

To validate the “Two-Hit” hypothesis, we grouped CAR and LAR by quartiles and plotted a heatmap of epilepsy incidence (Figure 3B). The results revealed a clear diagonal gradient in risk distribution: patients in the “Double Low” group (CAR-Q1 and LAR-Q1) had the lowest epilepsy incidence (2.4%), whereas those in the “Double High” group (CAR-Q4 and LAR-Q4) had the highest incidence (13.5%). Notably, the risk in the concurrent high-value group (13.5%) significantly exceeded the risk associated with the elevation of single markers (CAR-Q4 alone: ~5.5%; LAR-Q4 alone: ~4.3%). This strongly confirms that immune-inflammatory and hypoxia-metabolic stresses possess a significant synergistic effect in the epileptogenic process.

### Subgroup analysis and robustness

**Figure 4:**
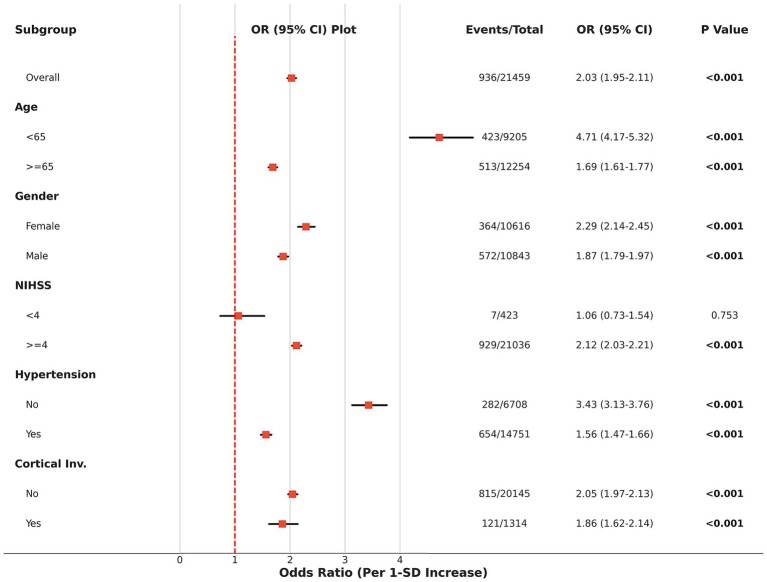
Subgroup analysis of the association between the immuno-nutritional index (CAR) and post-stroke epilepsy. Forest plot displaying the odds ratios (OR) for epilepsy risk per 1-standard deviation (SD) increase in CAR across prespecified clinical subgroups (Age, Gender, NIHSS, Hypertension, and Cortical Involvement). The red squares represent the point estimates of the OR, and the horizontal black lines indicate the 95% confidence intervals. The vertical dashed red line represents the reference value of OR = 1. The association between CAR and epilepsy remained significant and stable across all subgroups. NIHSS, National Institutes of Health Stroke Scale; CI, confidence interval.

To evaluate the robustness of CAR’s predictive value, we performed stratified subgroup analyses by age, gender, stroke severity (NIHSS), hypertension, and cortical involvement (Figure 4). The results indicated that the predictive value of CAR remained highly consistent across all subgroups (interaction *p*-values > 0.05). Clinically significant is the finding that even in subgroups with minor stroke (NIHSS < 4) and no cortical involvement, CAR remained a significant independent predictor. This suggests that the contribution of systemic immuno-nutritional dysregulation to epilepsy risk is independent of structural brain injury severity, implying that this marker holds important screening value even in traditionally low-risk populations.

### Incremental predictive value of the Two-Hit Model

**Figure 5:**
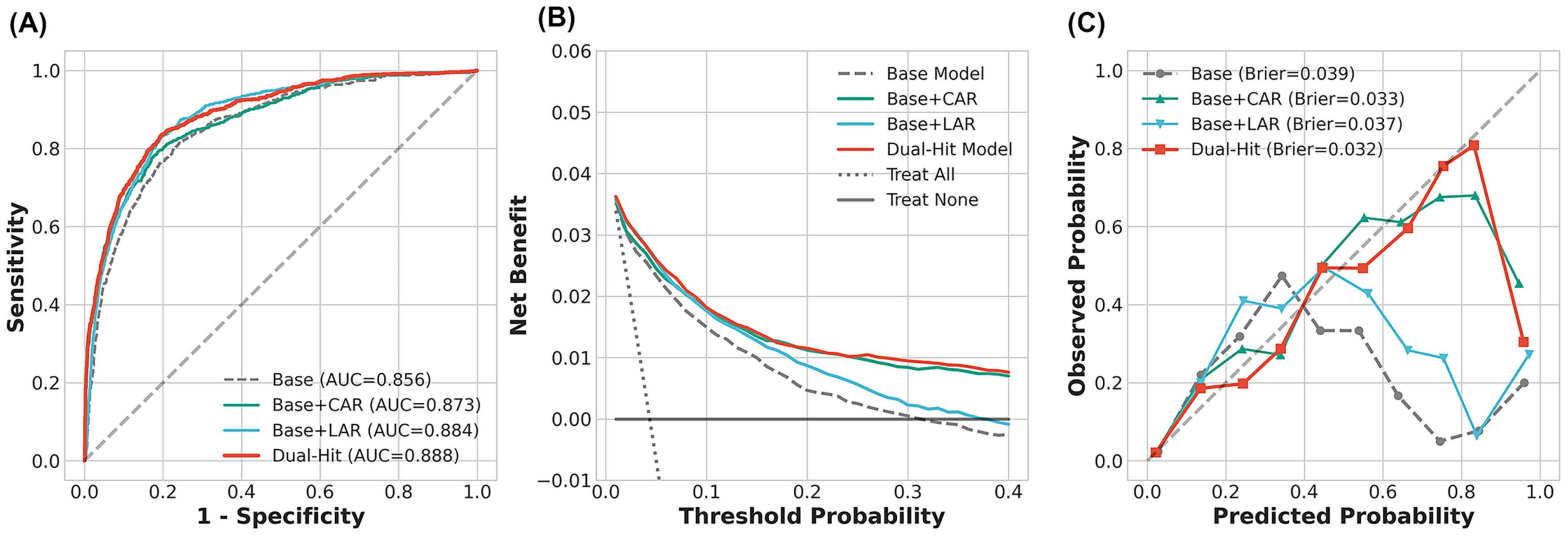
Comprehensive evaluation of the predictive performance of the “Two-Hit” model compared to baseline models. **(A)** Receiver operating characteristic (ROC) curves comparing the discrimination ability of the base model (clinical variables only), single-marker models (Base + CAR/LAR), and the Two-Hit model. The Two-Hit model achieved the highest area under the curve (AUC = 0.888). **(B)** Decision curve analysis (DCA) illustrating the clinical net benefit of the different models across a range of threshold probabilities. The Two-Hit model (red line) shows the highest net benefit. **(C)** Calibration curves assessing the agreement between predicted probabilities and observed outcomes. The Brier scores indicate the calibration accuracy, with the Two-Hit model showing the lowest score (0.032), indicating the best fit. AUC, area under the curve; CAR, C-reactive protein to albumin ratio; LAR, lactate to albumin ratio.

We further evaluated the improvement in prediction accuracy by adding CAR and LAR to traditional clinical models (Table 3; Figure 5). The Baseline Model, comprising demographic characteristics, NIHSS, and lesion location, yielded an AUC of 0.856 (95% CI 0.844–0.868). Adding single biomarkers improved model performance (Baseline + CAR: AUC 0.873; Baseline + LAR: AUC 0.884). The “Two-Hit Model,” integrating baseline features with both biomarkers, demonstrated the optimal discriminatory ability, achieving an AUC of 0.888 (95% CI 0.877–0.899) (Figure 5A). Beyond AUC enhancement, reclassification metrics showed substantial improvement in risk stratification: the Two-Hit Model yielded a NRI of 0.820 (95% CI 0.755–0.880, *p* < 0.001) and an IDI of 0.139 (95% CI 0.125–0.153, *p* < 0.001). DCA (Figure 5B) confirmed that the Two-Hit Model provided higher net benefit than the Baseline Model across a wide range of probability thresholds, and the calibration curve (Figure 5C) showed good agreement between predicted and observed probabilities (Brier score = 0.032).

**Table 3:** Incremental value of CAR and LAR for predicting post-stroke epilepsy.

Model	AUC (95% CI)	NRI (95% CI)	NRI *P*	IDI (95% CI)	IDI *P*
Base model	0.856 (0.844–0.868)	Ref	–	Ref	–
Base + CAR	0.873 (0.860–0.884)	0.611 (0.547–0.677)	<0.001	0.127 (0.112–0.142)	<0.001
Base + LAR	0.884 (0.873–0.894)	0.987 (0.936–1.038)	<0.001	0.040 (0.036–0.044)	<0.001
Two-Hit model	0.888 (0.877–0.899)	0.820 (0.755–0.880)	<0.001	0.139 (0.125–0.153)	<0.001

AUC, area under the curve; NRI, net reclassification index; IDI, integrated discrimination improvement; CI, confidence interval; CAR, C-reactive protein to albumin ratio; LAR, lactate to albumin ratio.

### Internal validation and calibration

To rigorously assess the robustness of the model, we performed internal validation using 1,000 bootstrap resamples. The model demonstrated high stability with minimal overfitting. As shown in Supplementary Table S5, the optimism-corrected AUC was 0.886, which was nearly identical to the apparent AUC (Optimism = 0.0004). Furthermore, the model exhibited excellent calibration, yielding an optimism-corrected Brier score of 0.033 and a calibration slope of 0.998. The calibration plot (Supplementary Figure S2) visually confirmed the strong agreement between predicted probabilities and observed outcomes across all risk deciles.

### Establishment and application of the web-based risk calculator

**Figure 6:**
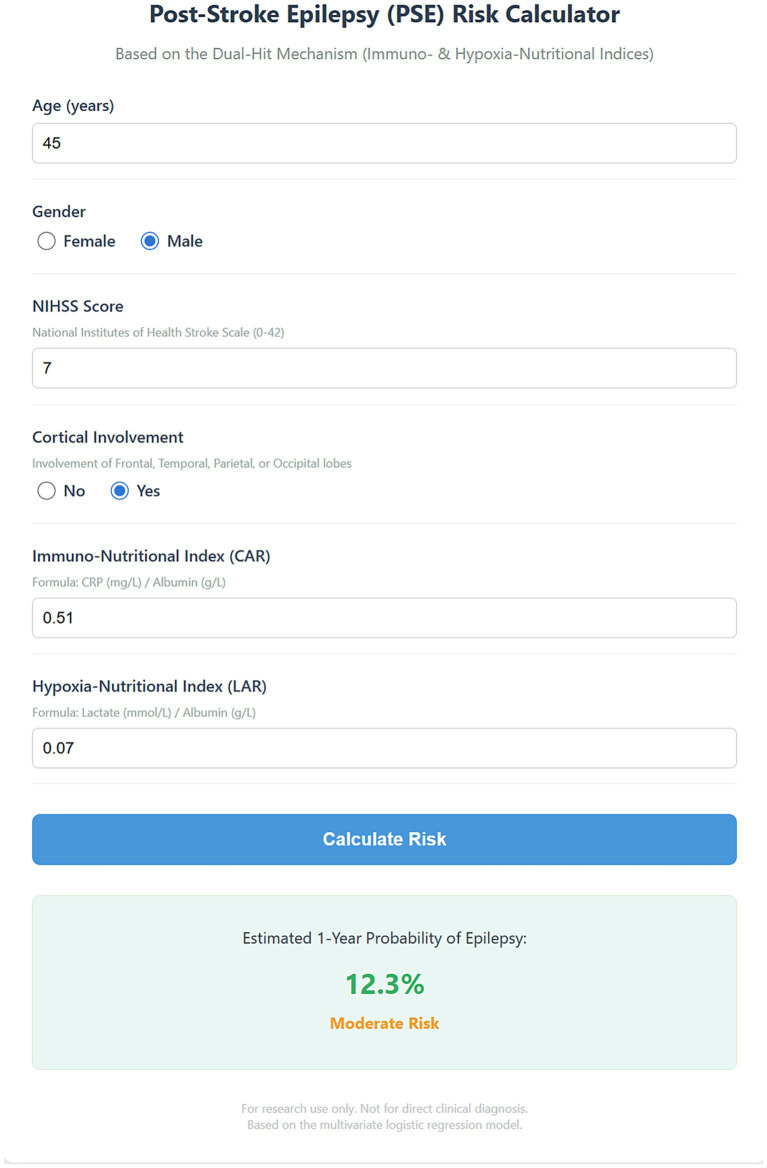
Interface of the web-based interactive risk calculator for post-stroke epilepsy. A screenshot of the digital tool developed based on the multivariate “Two-Hit” logistic regression model. The calculator integrates clinical variables (age, gender, NIHSS score, cortical involvement) with the two biomarkers (CAR and LAR) to generate a personalized 1-year probability of epilepsy. The example displays a patient with a calculated probability of 12.3%, categorized as “moderate risk.” NIHSS, National Institutes of Health Stroke Scale; CAR, C-reactive protein to albumin ratio; LAR, lactate to albumin ratio.

To facilitate the clinical translation and practical application of our findings, we constructed an interactive, open-access web-based risk calculator based on the final multivariate “Two-Hit” model (Supplementary material). This digital tool integrates easily accessible clinical variables (age, gender, NIHSS score, cortical involvement) and the two core biomarkers (CAR and LAR). Clinicians need only input the patient’s specific parameters, and the calculator instantly outputs the personalized probability of developing PSE within the next year (e.g., the example patient in Figure 6 has a predicted probability of 12.3%, classified as intermediate risk). Based on preset thresholds, the tool stratifies patients into risk categories (low/intermediate/high). Compared to traditional static nomograms, this dynamic tool avoids manual reading errors and enables rapid, precise bedside risk assessment to assist clinicians in formulating individualized neuroprotective and monitoring strategies.

## Discussion

In this large-scale retrospective cohort study of 21,459 patients with AIS, we proposed and validated a novel “Two-Hit” hypothesis involving immune-inflammatory and hypoxia-metabolic mechanisms in the pathogenesis of PSE. Our findings provide several key insights: First, even after rigorous adjustment for classic anatomical risk factors such as stroke severity (NIHSS) and cortical involvement, the Immuno-Nutritional Index (CAR) and Hypoxia-Nutritional Index (LAR) remained potent independent predictors of PSE. Second, these biomarkers exhibited complex non-linear dose–response relationships with epilepsy risk (J-shaped for CAR, bell-shaped for LAR). Third, a significant synergistic effect exists between the two, with the risk conferred by the “Two-Hit” far exceeding the sum of individual factors. Finally, incorporating these indices into clinical models significantly improved risk reclassification (NRI > 0.8), and our web-based risk calculator developed based on these findings provides a precise translational tool for identifying “metabolically high-risk” patients.

### Biological mechanisms of the “Two-Hit”: bridging systemic biomarkers to central pathology

The core innovation of this study lies in utilizing CAR and LAR to quantify the systemic “Two-Hit” effect. We explicitly interpret these acute-phase biomarkers not merely as statistical predictors, but as accessible systemic surrogates for the central pathological processes of “epileptogenic priming”—specifically, BBB disruption, neuroimmune activation, and cerebral metabolic dysregulation.

The First Hit (Immune-Inflammatory Cascade): We observed a robust J-shaped association between CAR and PSE risk. To address the temporal latency between acute inflammation and delayed seizures, we frame CAR not as a direct real-time driver of chronic epilepsy, but as a “risk-associated marker” reflecting the magnitude of the initial “epileptogenic priming.” Mechanistically, this acute-to-chronic bridge is mediated by early blood–brain barrier (BBB) dysfunction. Systemic inflammation (surged CRP) correlates strongly with circulating pro-inflammatory mediators such as IL-1β, TNF-α, and HMGB1, which can directly compromise endothelial tight junctions (13, 26). This acute leakage allows serum albumin to extravasate into the brain parenchyma. Crucially, unlike a passive bystander, this extravasated albumin binds to TGF-β receptors on perivascular astrocytes, triggering a persistent signaling cascade. This pathway leads to the downregulation of inwardly rectifying potassium channels (Kir4.1) and glutamate transporters (GLT-1), resulting in impaired potassium buffering and aberrant synaptogenesis that unfolds over weeks to months (10, 27–30). Therefore, although CAR is measured acutely, it captures the severity of the initial neuroimmune activation and BBB insult that sets the biological trajectory for delayed epileptogenesis. The sharp rise in risk when CAR exceeds the population mean (0 SD) implies that the epileptogenic network may only be fully activated when the inflammatory burden breaches the body’s compensatory limit.

### The second hit (hypoxia-metabolic stress)

The “bell-shaped” relationship between LAR and PSE provides novel insights into cerebral metabolic distress. Lactate is not merely a byproduct of anaerobic glycolysis but a critical modulator of neuronal excitability (14, 15). In the context of stroke, elevated lactate signals mitochondrial dysfunction and an “energy crisis” in the ischemic penumbra. The LAR index quantifies this metabolic stress relative to the host’s homeostatic reserve. Moderate elevation of LAR (approx. 0.10 SD) likely reflects a “metabolic hyper-excitatory state”: hypoxia-induced acidosis promotes glutamate release while inhibiting GABAergic transmission, thereby lowering the seizure threshold (31). However, the declining risk trend at extremely high LAR levels (>4 SD) suggests complex mechanisms. Biologically, severe acidosis might inhibit NMDA receptor activity (“paradoxical anticonvulsant” effect) or be accompanied by multiple organ failure, introducing a competing risk of death (32–34). Thus, LAR functions as a surrogate for the cerebral metabolic dysregulation that precedes network reorganization. Clinicians should maintain high vigilance for patients in the ‘moderate metabolic disturbance’ zone (LAR ~0.10 SD), where hypoxic metabolism is sufficient to induce excitotoxicity but has not yet reached the acidosis threshold for neural suppression.

Our heatmap analysis visually confirmed the “Two-Hit” hypothesis. Patients in the concurrent high-value group (High CAR + High LAR) had an epilepsy incidence (13.5%) nearly triple that of the single-marker elevation groups. This supports a pathological model where an inflammatory response and hypoxic hit, superimposed on a foundation of nutritional depletion (low albumin) constituting a susceptible brain microenvironment, produce cumulative damage that ultimately facilitates the formation of epileptogenic foci.

### Clinical translation: from anatomical prediction to metabolic functional assessment

Conventional wisdom suggests that PSE prediction relies primarily on neuroimaging anatomical features such as infarct location (35–37). However, subgroup analysis in this study revealed a clinically significant finding: CAR and LAR maintained significant predictive efficacy even in patients with minor stroke (NIHSS score < 4) and no cortical involvement (i.e., deep or subcortical infarcts). This result revises the traditional perception that “minor stroke or deep infarction implies low epilepsy risk,” indicating that systemic immuno-metabolic microenvironmental dysregulation can drive epileptogenesis independently of the extent of structural brain injury. For these “cryptic” patients with mild radiographic injury but hematological markers suggesting “high metabolic risk,” the model constructed in this study provides a crucial supplement for risk identification.

To promote the effective translation of statistical models into clinical application, we developed the complex multivariate regression model into an interactive web-based risk calculator. Compared to traditional static nomograms, this digital tool demonstrates significant clinical advantages: it enables immediate bedside assessment, provides precise individualized probability estimates, and effectively eliminates errors associated with manual chart reading. Using this tool, clinicians can rapidly identify patients without obvious cortical structural damage but with extremely high metabolic risk, thereby tailoring individualized monitoring plans (e.g., long-term EEG monitoring) or preventive neuroprotective strategies. The NRI of 0.820 further confirms the tool’s core value in optimizing risk stratification—specifically, accurately correcting misclassifications by traditional anatomical models and identifying potential high-risk patients within the low-risk population.

Furthermore, the findings of this study are not limited to risk stratification but also provide a new theoretical perspective for early intervention in PSE. Previous neurocritical care management has often focused on intracranial pressure and glycemic control, without fully appreciating the central role of basal nutritional status in neuro-immune regulation. Given that CAR and LAR reveal the critical position of “immuno-nutritional” imbalance in the epileptogenic process, targeted nutritional support strategies show potential value in preventing PSE. For instance, early initiation of enteral nutrition to maintain intestinal barrier integrity, or appropriate supplementation of human serum albumin in patients with severe hypoalbuminemia to restore the body’s antioxidant defense capacity, may help mitigate the pathological cascade of the “Two-Hit.” Although this hypothesis requires further verification through prospective randomized controlled trials, our model emphasizes the importance of integrating “nutritional resuscitation” into neuroprotective strategies, aligning closely with the “Immunonutrition” therapeutic concept currently advocated in critical care medicine (38, 39).

## Strengths and limitations

The primary strength of this study lies in its reliance on a large-scale multi-center cohort (*N* = 21,459), providing high statistical power that not only ensured model robustness but also allowed for the precise quantification of non-linear relationships and synergistic effects between CAR and LAR. Second, we transcended simple prediction by endowing routine laboratory metrics with new pathophysiological significance, demonstrating the feasibility of utilizing existing data to identify dysregulation in the neuro-immuno-metabolic network. Additionally, the web-based risk calculator we developed successfully translates complex statistical models into an intuitive clinical tool, facilitating the precise identification of cryptic patients with minor radiographic findings but high metabolic risk, thus possessing direct clinical translational value.

However, several limitations must be fully considered when interpreting the results.

First, this study is essentially an observational study based on clinical big data. While our statistical associations strongly support the “Two-Hit” hypothesis, molecular mechanisms linking elevated CAR and LAR to BBB disruption or neuronal excitotoxicity have not been directly verified via animal models or *in vitro* cellular experiments. Consequently, this study primarily provides epidemiological evidence bridging clinical phenomena and pathological concepts; specific molecular crosstalk mechanisms require further elucidation through basic research.

Second, it must be acknowledged that as systemic surrogate biomarkers for immune and metabolic status, CAR and LAR possess inherent limitations in specificity. Although we strictly adjusted for multiple confounders statistically, peripheral blood lactate and CRP levels may still be subject to non-specific interference from infections, shock, or other systemic comorbidities, and thus may not perfectly map localized microenvironmental changes in the brain parenchyma. Nevertheless, given the high accessibility and routine nature of these markers in emergency clinical practice, their comprehensive characterization of the organism’s pathophysiological microenvironment effectively balances the limitation of relative lack of specificity at the level of clinical application.

Third, the temporal resolution of our biomarker assessment was limited to a single time point upon admission. As dynamic acute-phase reactants, CRP and lactate levels fluctuate throughout the clinical course. While our data suggest that the initial magnitude of metabolic-inflammatory stress provides significant prognostic value—potentially by reflecting the severity of the initial “epileptogenic priming”—we could not evaluate whether persistent chronic inflammation or longitudinal biomarker trajectories further modify epilepsy risk. Therefore, these baseline metrics should be interpreted as risk-associated markers reflecting the initial insult severity rather than dynamic predictors of the evolving epileptogenic process. Future studies with serial measurements are warranted to characterize the temporal kinetics of these biomarkers.

Fourth, regarding the statistical modeling approach, we acknowledge that a competing risk model (e.g., Fine-Gray) would theoretically be superior for analyzing post-stroke outcomes where mortality is a significant competing event. We recognize that 813 patients were excluded due to early mortality or loss to follow-up; however, due to the constraints of this secondary analysis, detailed time-to-event data for these specific excluded cases were unavailable, precluding the execution of a competing risk analysis. Consequently, the risk probabilities generated by our logistic regression model should be interpreted as the “conditional probability” of developing epilepsy given that the patient has survived the acute phase of stroke. This limitation is particularly pertinent to interpreting the “bell-shaped” curve of LAR: the paradoxical decline in epilepsy risk at extremely high lactate levels likely reflects a survival bias, where critically ill patients face a competing risk of early mortality that precedes the onset of seizures. Therefore, we advise clinicians to interpret the predictive value of LAR with caution in this extreme range, understanding that the “lower” predicted risk may mask a fatal outcome.

Finally, regarding generalizability, we explicitly acknowledge that the lack of external validation is a significant limitation. While external validation—whether temporal or geographic—is mandatory for recommending an algorithm for routine bedside use, the fully anonymized nature of the public dataset precluded the extraction of hospital-specific or chronological metadata required for such analyses. To mitigate this, we performed rigorous internal validation using 1,000 bootstrap resamples, which demonstrated high model stability and minimal overfitting. However, strictly adhering to safety standards for medical algorithms, we emphasize that the current model serves primarily as a preliminary risk-stratification tool. Future prospective, multi-center studies are warranted to fully validate its performance across diverse populations before it is adopted as a clinical decision-support system.

## Conclusion

In summary, this study demonstrates that the Immuno-Nutritional (CAR) and Hypoxia-Nutritional (LAR) indices are potent synergistic predictors of PSE. They reveal a “Two-Hit” mechanism transcending mere anatomical structural injury, wherein inflammatory responses and metabolic stress drive epileptogenesis against a background of compromised nutritional defense. The web-based risk calculator we developed successfully translates this mechanism into a quantifiable scoring system. However, we emphasize that this calculator currently serves as a preliminary screening tool. While the model demonstrates robust internal validity, external validation in independent cohorts is mandatory before widespread clinical adoption. Therefore, at this stage, this algorithm should be utilized to support risk stratification strictly in a research context rather than as a sole determinant for altering treatment strategies. Beyond its immediate predictive utility, these findings provide a theoretical basis for future randomized controlled trials on targeted “nutrition-metabolic” interventions, suggesting that optimizing immuno-nutritional management in the acute phase of stroke may represent a novel strategy for preventing secondary epilepsy.

## Data Availability

The datasets presented in this study can be found in online repositories. The names of the repository/repositories and accession number(s) can be found at: The datasets analyzed during the current study are available in the Dryad repository, https://doi.org/10.5061/dryad.w0vt4b92c.
