# Supplementary material for: Synergistic impact of immuno-nutritional and hypoxia-metabolic disturbances on post-stroke epilepsy: a “Two-Hit” prediction model and web-based risk calculator
Source: Front Nutr. 2026 Feb 26;13:1759899. doi: 10.3389/fnut.2026.1759899 (PMC12979162; doi:10.3389/fnut.2026.1759899)
Supplement: Supplementary file 1 [file Data_Sheet_1.zip › Supplementary Material_calculator_v2.html]

Post-Stroke Epilepsy Risk Calculator


# Post-Stroke Epilepsy (PSE) Risk Calculator

## Based on the Dual-Hit Mechanism (Immuno- & Hypoxia-Nutritional Indices)

Age (years)

Gender

Female
 Male

NIHSS Score

National Institutes of Health Stroke Scale (0-42)

Cortical Involvement

Involvement of Frontal, Temporal, Parietal, or Occipital lobes

No
 Yes

Immuno-Nutritional Index (CAR)

C-reactive protein (mg/L) / Albumin (g/L) Ratio

Hypoxia-Nutritional Index (LAR)

Lactate (mmol/L) / Albumin (g/L) Ratio

Calculate Risk

Estimated 1-Year Probability of Epilepsy:

### 0.0%

For research use only. Not for direct clinical diagnosis.  
Based on the multivariate logistic regression model.
