# Supplementary material for: Synergistic impact of immuno-nutritional and hypoxia-metabolic disturbances on post-stroke epilepsy: a “Two-Hit” prediction model and web-based risk calculator
Source: Front Nutr. 2026 Feb 26;13:1759899. doi: 10.3389/fnut.2026.1759899 (PMC12979162; doi:10.3389/fnut.2026.1759899)
Supplement: Supplementary file 3 [file Table_2.docx]

**Supplementary Table 2.** The subsequent steps for use of the PROBAST+AI tool, conform the use of the original PROBAST tool.

Cite as: Moons KGM, Damen JAA, Kaul T, et al. PROBAST+AI: an updated quality, risk of bias, and applicability assessment tool for prediction models using regression or artificial intelligence methods. *BMJ* 2025;388:e082505 doi:10.1136/bmj-2024-082505

| **Step** | **Task** | **When to complete** |
| --- | --- | --- |
| 1 | Specify the intended purpose of the prediction model assessment or of the prediction model systematic review | Once per assessment or systematic review |
| 2 | Classify the type of prediction model study (development or evaluation or both) | Once for each prediction model of interest in each publication assessed, for each relevant outcome |
| 3 | Assess quality and applicability to the intended purpose of the developed prediction model, for each domain  &  Assess risk of bias and applicability to the intended purpose of the evaluated prediction model, for each domain | Once for each model development for each distinct prediction model in a publication  Once for each model evaluation for each distinct prediction model in a publication |
| 4 | Assess the overall quality and applicability for model development, and separately, the risk of bias and applicability for model evaluation | Once for each assessed prediction model in a publication, and separately for model development and for model evaluation |

Directly based on PROBAST-2019 ([www.probast.org).](http://www.probast.org/) We strongly recommend reading the Explanation and Elaboration Light (Supplementary Table 4) and the Explanation and Elaboration document of PROBAST-2019[^1^.](#_bookmark0) We also recommend checking the example papers on [www.probast.org.](http://www.probast.org/)

^1^ Moons KGM, Wolff RF, Riley RD, Whiting PF, Westwood M, Collins GS, et al. PROBAST: A Tool to Assess Risk of Bias and Applicability of Prediction Model Studies: Explanation and Elaboration. Ann Intern Med. 2019;170(1):W1- w33

## Step 1: Use the PICOTS guidance to specify your intended purpose or aim of the prediction model assessment, and the review question in case a systematic review of prediction model studies is conducted

PICOTS:

| **Item** | **Explanation** |
| --- | --- |
| **Population** | Define the target population (e.g., patients) in whom the assessed prediction models are to be applied. The target population not only directs search strings and in/exclusion criteria of prediction models or prediction model studies in  case of a systematic literature review, but also directs the applicability assessment. |
| **Index model(s)** | Define the targeted prediction models to be assessed, which may be a single prediction model (the index model) of which the predictive accuracy is meta- analysed across multiple external evaluation studies of that index model but may also address multiple prediction models (developed or evaluated) for the  targeted population, outcome or setting, depending on the assessor’s or prediction model review focus. |
| **Comparator model(s)** | Define the other prediction models whose predictive ability is compared to that of the index model. |
| **Outcome(s)** | Define the outcomes or endpoints that are predicted by the index (and possibly comparator) prediction models in the target population. |
| **Timing** | 1. Define the moment or time-point (e.g., in the patient work-up) at which the prediction with the prediction models is made (i.e., the start point or T0 of the use of the models). 2. Define the time or follow-up period in which the outcomes are being predicted by the prediction models in the targeted population (prediction   horizon). |
| **Setting and intended use of the prediction model** | Define the healthcare setting or context to which the index prediction models  apply. The prediction ability of models may change across healthcare settings or contexts. |

*The following table should be completed once per prediction model review.*

| **Population** | Patients with Acute Ischemic Stroke (AIS). |
| --- | --- |
| **Index model(s)** | "Two-Hit" Prediction Model / Web-Based Risk Calculator. |
| **Comparator model(s)** | Baseline Model (Clinical features only) & Single-Marker Models. |
| **Outcome(s)** | Post-Stroke Epilepsy (PSE) within 1 year. |
| **Timing** | At admission (T0), predicting 1-year risk. |
| **Setting and intended use of the prediction model** | Hospital setting / Clinical use. |

## Step 2: Classify the type of prediction model assessment

Use the following table to classify the assessments as model development, model evaluation, or combination (see also Box 1 in the paper). Different signalling questions apply for different types of prediction model assessments. If the assessment does not fit one of these classifications, then PROBAST+AI should not be used.

| **Classify the assessment based on its aim** | | | |
| --- | --- | --- | --- |
| *Type of prediction study* | *Explanation* | *PROBAST+AI boxes to complete* | *Tick as appropriate* |
| Development only | Prediction model development only, i.e., without evaluation of its performance. | Model development |  |
| Evaluation only | External validation of one or more existing models in new data | Model evaluation |  |
| Combination | Prediction model development combined in the same study (publication) with the evaluation of its apparent performance, internal validation performance, or external validation performance. | Model development and evaluation | ☑  The study developed the model and evaluated its performance (apparent & internal validation via bootstrap) in the same dataset. |

## Step 3: Assess quality and applicability or risk of bias and applicability

This table should be completed once for each publication being assessed and for each relevant outcome in the prediction model review

| **Publication reference** | / |
| --- | --- |
| **Models of interest** | / |
| **Outcome of interest** | / |

# PROBAST+AI: MODEL DEVELOPMENT

Each domain of the model development part is judged for concerns about quality (low, high, or unclear concern). Each domain includes signalling questions to help make judgements. Signalling questions are rated as yes (Y), probably yes (PY), probably no (PN), no (N), no information (NI), and in some cases not applicable (NA, items marked with an asterisk*). All signalling questions are phrased so that ‘yes’ or ‘probably yes’ indicate low concerns for quality (= high quality).

Any signalling question rated as ‘no’ or ‘probably no’ flags the potential for sincere concerns regarding quality of that domain. You will need to use your judgement to determine whether the entire domain should be rated as ‘high’, ‘low’, or ‘unclear’ concern regarding quality. The Explanation & Elaboration Light (Supplementary Table 4) contains further information and examples on rating signalling questions and concerns regarding quality for each domain of the model development process.

The first three domains are also rated for concerns regarding applicability (low/high/unclear) of the prediction model (study) to the review question or to intended use of the assessed prediction models (as defined in step 1).

| **DOMAIN 1: Participants and data sources** | |
| --- | --- |
| **A. Quality** | |
| *Describe the sources of data and criteria for participant selection: Content: Data sourced from the Dryad Digital Repository (doi:10.5061/dryad.w0vt4b92c), originally from a multi-center retrospective cohort in Chongqing, China (2013-2022). Inclusion: AIS confirmed by CT/MRI. Exclusion: History of epilepsy, hemorrhagic stroke, etc.* | |
|  | **Y/ PY/ PN/ N/ NI** |
| **1.1 Were appropriate data sources used?** | Y |
| **1.2 Was an appropriate study design used?** | Y |
| **1.3 Did the in- and exclusions of study participants result in a representative**  **dataset?** | Y |
| **Concern regarding quality of selection of participants and data sources** | **QUALITYCONCERN:**  ***low/high/unclear*** |
| ***Rationale of quality rating:***   - 1. ***Data sourced from a publicly available, high-quality repository (Dryad), originating from a multi-center cohort study.***   2. ***Retrospective cohort study design is appropriate for prognostic model development.***   3. ***Inclusion criteria (AIS confirmed by CT/MRI) and exclusions (history of epilepsy, etc.) are standard and result in a representative AIS population.*** | |
| **B. Applicability** | |
| Describe included data sources, participants, setting, and dates: 21,459 AIS patients from multiple centers in China. Setting: Acute hospital admission. | |
| **Concern that the (data of the) included participants do not match the review question or the assessor’s intended use of the prediction model** | **APPLICABILITY CONCERN:**  ***low*** |

***Rationale of applicability rating:*** ***The participants match the target population (AIS patients) of the review question.***

| **DOMAIN 2: Predictors** | |
| --- | --- |
| **A. Quality** | |
| *List and describe predictors included in the final prediction model, how they were defined and assessed, and their timing of assessment:* *C-reactive protein to Albumin Ratio (CAR), Lactate to Albumin Ratio (LAR), NIHSS score, Age, Gender, Cortical Involvement. Measured at admission.* | |
|  | **Y/ PY/ PN/ N/ NI** |
| **2.1 Were predictors defined and assessed in a similar way for all participants?** | Y |
| **2.2 Was any pre-processing of predictors similar for all participants?** | Y |
| **2.3 Were predictor assessments made without knowledge of outcome data?** | Y |
| **2.4 Were the predictors included in the model available at the time the model**  **was intended to be used?** | Y |
| **Concern regarding the quality of the predictors or their assessment** | **QUALITY CONCERN:**  ***low*** |
| ***Rationale of quality rating:***  ***2.1 Predictors are standard laboratory values (CRP, Albumin, Lactate) and clinical scales (NIHSS), assessed uniformly at admission.***  ***2.2 Ratios (CAR, LAR) were calculated using standard formulas for all patients. Standardization (Z-score) was applied uniformly.***  ***2.3 Predictors were measured at admission, chronologically prior to the outcome (1-year epilepsy).***  ***2.4 All predictors are routine admission data available in acute clinical settings.*** | |
| **Applicability** | |
| **Concern that the definition, pre-processing, assessment, or timing of assessment of the predictors in the model do not match the review question or**  **the assessor’s intended use** | **APPLICABILITY CONCERN:**  ***low*** |
| ***Rationale of applicability rating:*** ***All predictors are routine clinical markers available in standard hospital settings.*** | |

| **DOMAIN 3: Outcome** | |
| --- | --- |
| **A. Quality** | |
| *Describe the outcome, how it was defined and determined, and the time interval between predictor assessment and outcome determination: Post-Stroke Epilepsy (PSE), defined as seizures occurring within 1 year of follow-up. Ascertained from medical records.* | |
|  | **Y/ PY/ PN/ N/ NI** |
| **3.1 Were outcomes defined and assessed appropriately?** | Y |
| **3.2 Were outcomes defined and assessed in a similar way for all participants?** | Y |
| **3.3 Were outcome assessments made without use or knowledge of predictor data?** | PY |
| **3.4 Was the time interval between predictor assessment and outcome assessment appropriate?** | Y |
| **Concern regarding quality of the outcome or its determination** | **QUALITY CONCERN:**  ***low*** |
| ***Rationale of quality rating:***  ***3.1 PSE defined as seizures occurring within 1 year is a standard clinical definition.***  ***3.2 Follow-up protocol appears consistent across the cohort.***  ***3.3 Although retrospective, epilepsy is an objective clinical event, unlikely to be biased by knowledge of admission albumin/lactate levels.***  ***3.4 1-year follow-up is sufficient to capture early and delayed PSE.*** | |
| **B. Applicability** | |
| *At what time point was the outcome determined:*  *If a composite outcome was used, describe the relative frequency/distribution of each contributing outcome:* | |
| **Concern that the outcome, its definition, assessment, or timing of assessment do not match the review question or the assessor’s intended use** | **APPLICABILITY CONCERN:**  ***low*** |
| ***Rationale of applicability rating:*** ***Matches the clinical question (1-year PSE risk).*** | |

| **DOMAIN 4: Analysis** | |
| --- | --- |
| **Quality** | |
| *Describe the numbers of participants, number of candidate predictors, number of outcome events:* *N = 21,459 participants. Outcomes = 936 (4.36%).* | |
| *Describe how the prediction model was developed (e.g., with respect to modelling technique, predictor selection, and classification or risk group definition):* *Multivariable Logistic Regression. Feature selection via LASSO and Random Forest. Restricted Cubic Splines (RCS) used for non-linear relationships.* | |
| *Describe the performance measures of the prediction model, e.g., (re)calibration, discrimination, (re)classification, net benefit, and whether they were adjusted for optimism:Yes.* | |
| *Describe missing data on predictors and outcomes as well as methods used for handling these missing data:* *Complete Case Analysis used. Figure 1 indicates exclusion of ~6,496 patients due to "missing important data".* | |
|  | **Y/ PY/ PN/ N/ NI/ NA** |
| **4.1 Was there evidence that the sample size was reasonable?** | Y |
| **4.2 Were continuous and categorical predictors handled appropriately?** | Y |
| **4.3 Were participants with missing or censored data handled appropriately in the analysis?** | PN |
| **4.4 If methods to address class imbalance were used, was the model or the model predictions recalibrated?*** | NA |
| **4.5 Were methods used to address potential model overfitting?** | Y |
| **Concern regarding quality of the analysis** | **QUALITY CONCERN:**  ***unclear to high*** |
| ***Rationale of quality rating:***  ***4.1 N=21,459 with 936 events. EPV (Events Per Variable) is >100, far exceeding the minimum requirement (EPV>10-20).***  ***4.2 Used Restricted Cubic Splines (RCS) for non-linear relationships (CAR/LAR). Did not dichotomize continuous variables arbitrarily.***  ***4.3 Figure 1 shows "missing important data (6,496)" were excluded. This "Complete Case Analysis" excludes ~23% of potential participants, which can introduce bias (unless data are Missing Completely At Random). PROBAST recommends multiple imputation.***  ***4.4 Standard logistic regression was used; no mention of SMOTE or undersampling/oversampling for the regression model itself.***  ***4.5 Feature selection via LASSO/Random Forest. Internal validation via Bootstrap (1000 iterations) to correct for optimism.***  ***Note: Rated Unclear/High primarily due to Item 4.3 (handling of missing data). Other aspects are excellent.*** | |

# PROBAST+AI: MODEL EVALUATION

Each domain of the model evaluation part is judged for risk of bias (low, high, or unclear). Each domain includes signalling questions to help make judgements. Signalling questions are again rated as yes (Y), probably yes (PY), probably no (PN), no (N), no information (NI), and in some cases not applicable (NA, items marked with an asterisk*). All signalling questions are phrased so that ‘yes’ or ‘probably yes’ indicate low risk of bias. Any signalling questions rated as ‘no’ or ‘probably no’ flags the potential for high risk of bias in that domain. You will need to use your judgement to determine whether the entire domain should be rated as ‘low’, ‘high’, or ‘unclear’ risk of bias.

If investigators only performed an apparent performance evaluation of the model, the responses to the signalling questions of domain 1, 2, and 3 in the Model Development section, can directly be copied and pasted to domain 1, 2, and 3 of this Model Evaluation section. However, the risk of bias judgement in the estimated model performance measures (e.g., their calibration or discrimination) still needs to be made.

Domain 4 should be evaluated separately for each type of model evaluation/validation assessed (apparent performance, internal validation, external validation). Shaded boxes indicate where signalling questions do not apply and should not be answered. The Explanation & Elaboration Light (Supplementary Table 4) contains further information and examples on rating signalling questions and risk of bias for each domain of model evaluation.

The first three domains are also rated for concerns regarding applicability (low/high/unclear) of the prediction model (study) to the review question or the intended use of the assessed prediction model(s) (as defined in step 1).

***Statement: As this study represents a "Combination" analysis where model development and evaluation (internal validation via bootstrapping) were conducted on the same dataset, the participants, predictors, and outcome definitions for the evaluation phase are identical to those in the development phase. Therefore, the assessment of these domains mirrors the development section.***

| **DOMAIN 1: Participants and data sources** | |
| --- | --- |
| **A. Risk of bias** | |
| *Describe the sources of data and criteria for participant selection:* *Same as development. Evaluation was performed on the same dataset of 21,459 patients using bootstrapping.* | |
|  | **Y/ PY/ PN/ N/ NI** |
| **1.1 Were appropriate data sources used?** | Y |
| **1.2 Was an appropriate study design used?** | Y |
| **1.3 Did the in- and exclusions of study participants result in a representative**  **dataset?** | Y |
| **Risk of bias introduced by the selection of participants and data sources** | **RISK OF BIAS:**  ***low*** |
| ***Rationale of risk of bias rating:*** ***The evaluation was performed using internal validation (bootstrapping) on the original development dataset. Thus, the data sources and participant selection criteria are identical to the development phase, with no new selection bias introduced specifically for the evaluation step.*** | |
| **B. Applicability** | |
| Describe included data sources, participants, setting, and dates: Same as development (AIS patients, China). | |
| **Concern that the (data of the) included participants do not match the review question or the assessor’s intended use of the prediction model** | **APPLICABILITY CONCERN:**  ***lowr*** |
| ***Rationale of applicability rating:*** ***The evaluation was performed using internal validation (bootstrapping) on the original development dataset. Thus, the data sources and participant selection criteria are identical to the development phase, with no new selection bias introduced specifically for the evaluation step.*** | |

| **DOMAIN 2: Predictors** | |
| --- | --- |
| **A. Risk of bias** | |
| *List and describe predictors included in the evaluated model, e.g., definition and timing of assessment:* *Same as development (CAR, LAR, NIHSS, etc.).* | |
|  | **Y/ PY/ PN/ N/ NI** |
| **2.1 Were predictors defined and assessed in a similar way for all participants?** | Y |
| **2.2 Was any pre-processing of predictors similar for all participants?** | Y |
| **2.3 Were predictor assessments made without knowledge of outcome data?** | Y |
| **2.4 Were the predictors included in the model available at the time the model was**  **intended to be used?** | Y |
| **Risk of bias introduced by the predictors or their assessment** | **RISK OF BIAS:**  ***low*** |
| ***Rationale of risk of bias rating:*** ***Predictor definitions, measurement timing, and pre-processing (including the calculation of CAR/LAR ratios and standardization) were identical to those used in the model development phase. Assessments were made without knowledge of the outcome, as they were baseline admission data.*** | |
| **B. Applicability** | |
| **Concern that the definition, pre-processing, assessment, or timing of assessment of the predictors in the model do not match the review question or the assessor’s intended**  **use** | **APPLICABILITY CONCERN:**  ***low*** |
| ***Rationale of applicability rating:*** ***Predictor definitions, measurement timing, and pre-processing (including the calculation of CAR/LAR ratios and standardization) were identical to those used in the model development phase. Assessments were made without knowledge of the outcome, as they were baseline admission data.*** | |

| **DOMAIN 3: Outcome** | |
| --- | --- |
| **A. Risk of bias** | |
| *Describe the outcome, how it was defined and determined, and the time interval between predictor assessment and outcome determination:* *Same as development (PSE within 1 year).* | |
|  | **Y/ PY/ PN/ N/ NI** |
| **3.1 Were outcomes defined and assessed appropriately?** | Y |
| **3.2 Were outcomes defined and assessed in a similar way for all participants?** | Y |
| **3.3 Were outcome assessments made without use or knowledge of predictor data?** | Y |
| **3.4 Was the time interval between predictor assessment and outcome assessment appropriate?** | Y |
| **Risk of bias introduced by the outcome or its determination** | **RISK OF BIAS:**  ***low*** |
| ***Rationale of risk of bias rating:*** ***The outcome definition (Post-Stroke Epilepsy within 1 year) and assessment method remained consistent between the development and evaluation phases, as the validation was conducted on the same patient cohort.*** | |
| **B. Applicability** | |
| *At what time point was the outcome determined:*  *If a composite outcome was used, describe the relative frequency/distribution of each contributing outcome:* | |
| **Concern that the outcome, its definition, assessment, or timing of assessment do not match the review question or the assessor’s intended use** | **APPLICABILITY CONCERN:**  ***low*** |
| ***Rationale of applicability rating:*** ***The outcome definition (Post-Stroke Epilepsy within 1 year) and assessment method remained consistent between the development and evaluation phases, as the validation was conducted on the same patient cohort.*** | |

| **DOMAIN 4: Analysis** | | | |
| --- | --- | --- | --- |
| **Risk of bias** | | | |
| *Describe numbers of participants, number of predictors, outcome events and events per predictor:* *Validation performed on the cohort of N = 21,459 using 1,000 bootstrap resamples.* | | | |
| *Describe the performance measures of the evaluated model, e.g., (re)calibration, discrimination, (re)classification, net benefit, and whether they were adjusted for optimism:* *Optimism-corrected AUC, Calibration Slope, Brier Score, and Net Benefit (DCA) were calculated.* | | | |
| *Describe any participants who were excluded from the analysis:* *Participants with missing data were excluded prior to validation (inherited from development).* | | | |
| *Describe missing data on predictors and outcomes as well as methods used for handling these missing data:* *Complete Case Analysis used. Figure 1 indicates exclusion of ~6,496 patients due to "missing important data".* | | | |
|  | **Y/ PY/ PN/ N/ NI/ NA** | | |
| **4.1 Was model evaluation based on only apparent performance avoided?** |  | | |
|  | **A** | **I** | **E** |
| **4.2 Was there evidence that the sample size was reasonable?** | Y | Y | Y |
| **4.3 Were participants with missing or censored data handled appropriately in the analysis?** | PN | PN | PN |
| **4.4 If methods to address class imbalance were used, was the evaluation done in**  **a dataset without imbalance correction?*** | Y | Y | Y |
| **4.5 If data splitting was done to create training and test datasets, was there evidence that data leakage was avoided?*** | **NA** | NA | **NA** |
| **4.6 If resampling methods were used to evaluate model performance, were all**  **model development steps replicated in the resampling process?*** | **NA** | Y | **NA** |
| **4.7 Was the predictive performance of the model evaluated appropriately, e.g., calibration, discrimination, and net benefit?** | Y | Y | Y |
| **Risk of bias introduced by the analysis** | **RISK OF BIAS:**  ***unclear*** | | |
| ***Rationale of risk of bias rating:***  ***Rated Unclear instead of High because the sheer sample size (21k) mitigates some risks, but the "Missing Data" issue (4.3) prevents a "Low" rating.*** | | | |

**Step 4: Assess the overall concerns regarding quality, risk of bias and applicability of the prediction model**

Use the following tables to reach overall judgements about concerns regarding quality and applicability for the model development process, and separately about risk of bias and concerns regarding applicability for the model performance evaluation. Complete for each assessed model.

**Model development**

| **OVERALL CONCERN REGARDING QUALITY OF THE PREDICTION MODEL DEVELOPMENT** | |
| --- | --- |
| **Low concern regarding quality** | If all four domains were rated low concern regarding quality. |
| **High concern regarding quality** | If at least one domain was rated high concern regarding quality . |
| **Unclear concern regarding quality** | If at least one domain was rated unclear concern regarding quality and no domains were rated high concern. |

| **OVERALL CONCERN FOR APPLICABILITY OF THE PREDICTION MODEL DEVELOPMENT** | |
| --- | --- |
| **Low concern for applicability** | If all three domains were rated low concern for applicability. |
| **High concern for applicability** | If at least one domain was rated high concern for applicability. |
| **Unclear concern for applicability** | If at least one domain was rated unclear concern for applicability and no domains were rated high concern. |

**Model evaluation**

| **OVERALL RISK OF BIAS OF THE PREDICTION MODEL EVALUATION** | |
| --- | --- |
| **Low risk of bias** | If all four domains were rated low risk of bias. |
| **High risk of bias** | If at least one domain was rated high risk of bias. |
| **Unclear risk of bias** | If at least one domain was rated unclear risk of bias and no domains were rated high risk of bias. |

| **OVERALL CONCERN FOR APPLICABILITY OF THE PREDICTION MODEL EVALUATION** | |
| --- | --- |
| **Low concern for applicability** | If all three domains were rated low concern for applicability. |
| **High concern for applicability** | If at least one domain was rated high concern for applicability. |
| **Unclear concern for applicability** | If at least one domain was rated unclear concern for applicability and no domains were rated high concern. |

| **OVERALL JUDGEMENT OF PREDICTION MODEL** | |
| --- | --- |
| **Overall judgement of quality (development)** | **QUALITY CONCERN:**  *unclear* |
| *Summary of quality concern:* *Methodologically rigorous (RCS, Feature Selection), but handled missing data by exclusion, which is a potential bias.* | |
| **Overall judgement of risk of bias (evaluation)** | **RISK OF BIAS:**  *unclear* |
| *Summary of sources of potential bias:* *Internal validation used appropriate Bootstrap methods and reported comprehensive metrics (Optimism-corrected AUC, Calibration). However, the evaluation was conducted on the dataset that excluded missing cases, inheriting the bias from Domain 4.3.* | |
| **Overall judgement of applicability (development)** | **APPLICABILITY CONCERN:**  *low* |
| *Summary of applicability concern:* *Participants, predictors, and outcome align perfectly with the clinical question.* | |
| **Overall judgement of applicability (evaluation)** | **APPLICABILITY CONCERN:**  *low* |
| *Summary of applicability concern:* *The evaluated population matches the target population.* | |
