# Supplementary material for: Synergistic impact of immuno-nutritional and hypoxia-metabolic disturbances on post-stroke epilepsy: a “Two-Hit” prediction model and web-based risk calculator
Source: Front Nutr. 2026 Feb 26;13:1759899. doi: 10.3389/fnut.2026.1759899 (PMC12979162; doi:10.3389/fnut.2026.1759899)
Supplement: Supplementary file 4 [file Table_3.docx]

**Supplementary Files 2**

**Supplemental Figure S1** LASSO regression-based feature selection and Random Forest-based feature importance ranking for prediction of post-stroke epilepsy.

**Supplemental Figure S2** Apparent calibration curve of the final “Two-Hit” multivariable prediction model for post-stroke epilepsy.

**Supplementary Table S3.** Detailed Specifications of the Web-based Risk Calculator Model.

**Supplementary Table S4.** Assessment of Conceptual Independence: Multicollinearity Diagnosis and Sensitivity Analysis.

**Supplementary Table S5.** Internal Validation of the "Two-Hit" Prediction Model using Bootstrap Resampling.

**Supplemental Figure S1** LASSO regression-based feature selection and Random Forest-based feature importance ranking for prediction of post-stroke epilepsy.

**(A)** Standardized coefficients (absolute values) of variables retained in the LASSO regression model (λ chosen via 10-fold cross-validation). Gray bars represent standard clinical variables/covariates; red bars highlight the two novel immuno-nutritional and hypoxia-nutritional biomarkers (CAR and LAR). Larger absolute coefficients indicate stronger contribution to variable selection and shrinkage.

**(B)** Feature importance ranking derived from Random Forest model, measured by mean decrease in Gini impurity. Gray bars represent standard clinical variables/covariates; red bars indicate the novel biomarkers (CAR and LAR). Higher values reflect greater contribution to reducing node impurity and improving classification of post-stroke epilepsy outcome.

**Abbreviations:** NIHSS = National Institutes of Health Stroke Scale; WBC = white blood cell count; CAR = C-reactive protein to albumin ratio; LAR = lactate to albumin ratio; d_dimer = D-dimer; HbA1c = glycosylated hemoglobin.


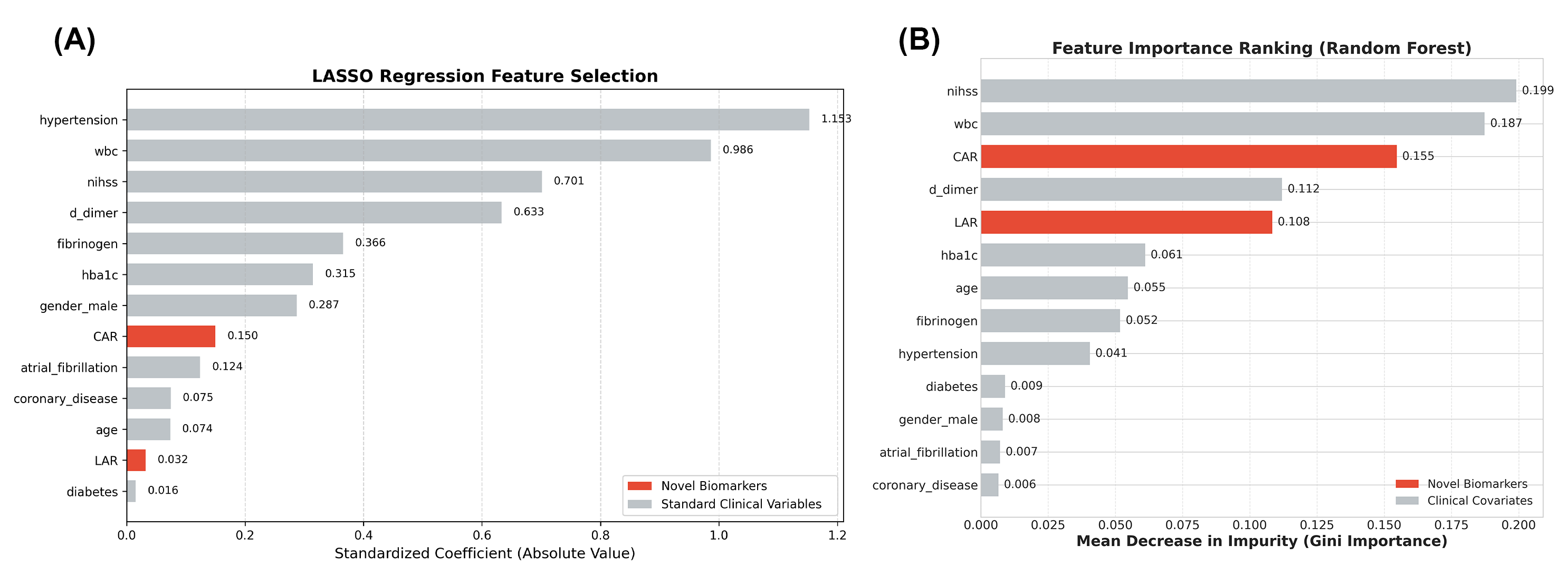


**Supplemental Figure S2** Apparent calibration curve of the final “Two-Hit” multivariable prediction model for post-stroke epilepsy.

The green squares and connecting line represent the apparent (non-optimism-corrected) calibration of the model across deciles of predicted risk. The dashed 45-degree line indicates perfect calibration (observed = predicted probability). The model demonstrates good overall calibration with minor underestimation at very low probabilities and slight overestimation in the mid-range; the corresponding area under the receiver operating characteristic curve (AUC) was 0.886.


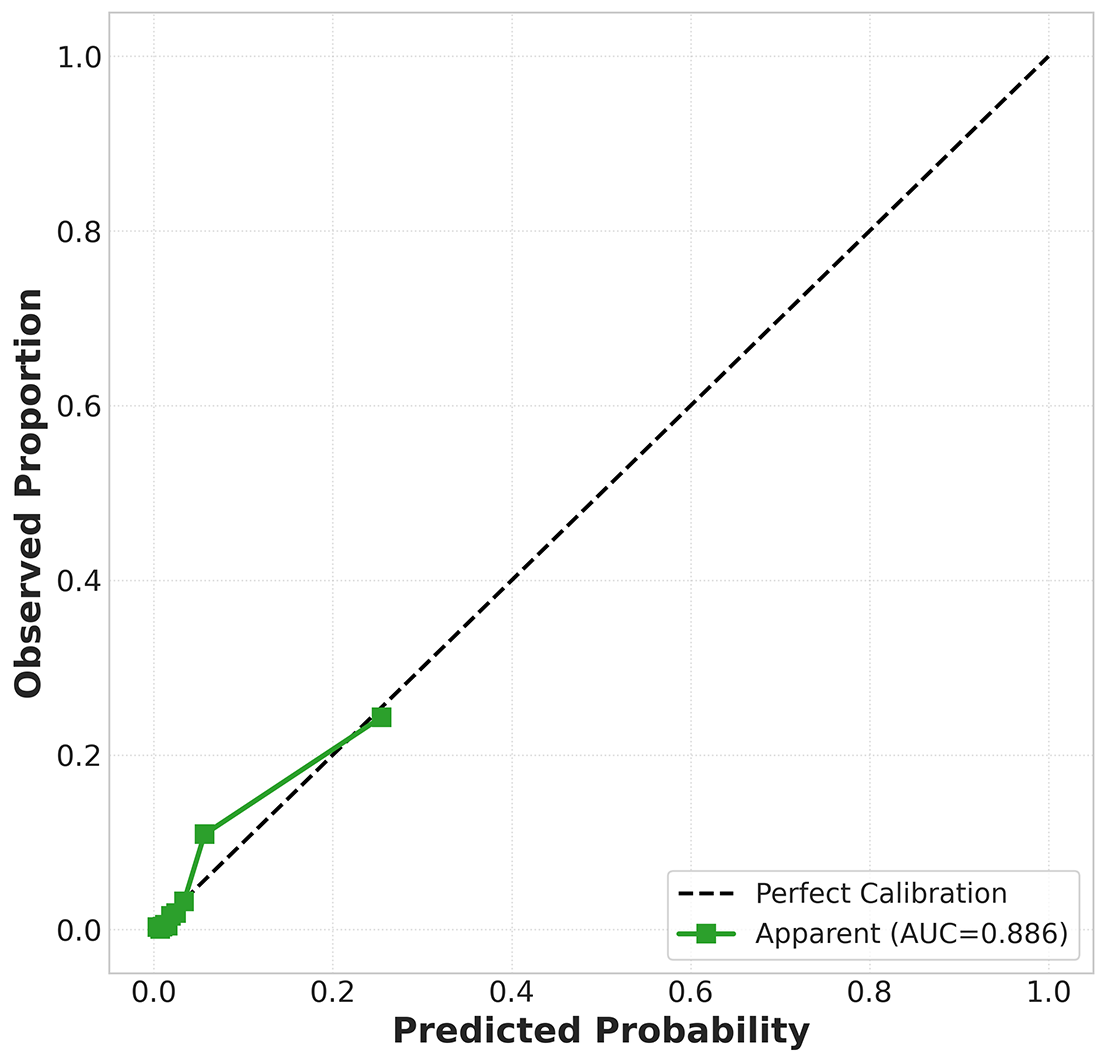


| **Supplementary Table S3. Detailed Specifications of the Web-based Risk Calculator Model.** | | | | | | |
| --- | --- | --- | --- | --- | --- | --- |
| Predictor | Coefficient (β) | Std. Error | z-value | P-value | Adjusted OR | 95% CI for OR |
| Intercept | -3.21 | 0.215 | -14.91 | <0.001 | — | — |
| Age (per year) | -0.048 | 0.003 | -16.04 | <0.001 | 0.95 | 0.95–0.96 |
| Gender (Male) | 0.64 | 0.082 | 7.85 | <0.001 | 1.9 | 1.62–2.22 |
| NIHSS Score (per point) | 0.27 | 0.012 | 22.88 | <0.001 | 1.31 | 1.28–1.34 |
| Cortical Involvement (Yes)^*^ | 0.49 | 0.117 | 4.19 | <0.001 | 1.64 | 1.30–2.06 |
| Immuno-Nutritional Index (CAR)^†^ | 0.51 | 0.024 | 20.83 | <0.001 | 1.66 | 1.58–1.74 |
| Hypoxia-Nutritional Index (LAR)^†^ | 0.39 | 0.028 | 13.84 | <0.001 | 1.48 | 1.40–1.57 |
| OR: Odds Ratio; CI: Confidence Interval; NIHSS: National Institutes of Health Stroke Scale; CAR: C-reactive protein to Albumin Ratio; LAR: Lactate to Albumin Ratio. * Cortical involvement is defined as a binary variable indicating the presence of acute infarction in the frontal, temporal, parietal, or occipital lobes. † CAR and LAR were standardized (Z-score transformed) before modeling. The Odds Ratios represent the change in risk per 1-standard deviation increase. | | | | | | |

**Supplementary Table S4.** Assessment of Conceptual Independence: Multicollinearity Diagnosis and Sensitivity Analysis.

**Panel A: Multicollinearity Diagnosis (Variance Inflation Factor)**

| Biomarker Index | VIF^∗^ | Interpretation |
| --- | --- | --- |
| Immuno-Nutritional Index (CAR) | 1.42 | No significant multicollinearity |
| Hypoxia-Nutritional Index (LAR) | 1.12 | No significant multicollinearity |
| Other covariates (e.g., Age, NIHSS) | < 2.0 | No significant multicollinearity |

VIF: Variance Inflation Factor; CAR, C-reactive protein to Albumin Ratio; LAR, Lactate to Albumin Ratio; NIHSS: National Institutes of Health Stroke Scale

∗A VIF value < 5 indicates that the variable is not subject to significant multicollinearity. Values are based on the full multivariate model including all adjusted covariates.

**Panel B: Sensitivity Analysis (Model Performance Comparison)**

| Model Strategy | Key Biomarkers Included | AUC | AIC | BIC |
| --- | --- | --- | --- | --- |
| Model A: "Decomposed" Model | CRP + Lactate + Albumin^†^ | 0.892 | 5359.8 | 5455.5 |
| Model B: "Two-Hit" Ratio Model | CAR + LAR | 0.888 | 5431.5 | 5519.3 |

AUC, Area Under the Curve; AIC, Akaike Information Criterion; BIC, Bayesian Information Criterion; CAR, C-reactive protein to Albumin Ratio; LAR, Lactate to Albumin Ratio.

† Modeled as separate covariates alongside standard clinical adjusters (age, gender, NIHSS, cortical involvement). Note: The comparable AUC values (0.892 vs. 0.888) indicate that the composite ratio indices effectively capture the synergistic prognostic information of their constituent components without substantial information loss.

| **Supplementary Table S5.** Internal Validation of the "Two-Hit" Prediction Model using Bootstrap Resampling. | | | |  |
| --- | --- | --- | --- | --- |
| Performance Metric | Apparent Performance (95% CI) | Bootstrap Optimism^*^ | Optimism-Corrected Performance^†^ | Ideal Value |
| Discrimination |  |  |  |  |
| Area Under the ROC Curve (AUC) | 0.886 (0.875–0.897) | 0.0004 | 0.886 | 1 |
| Calibration |  |  |  |  |
| Brier Score | 0.033 | < 0.001 | 0.033 | 0 |
| Calibration Slope | 1 | 0.002 | 0.998 | 1 |
| Calibration Intercept | 0 | 0.008 | -0.008 | 0 |
| * Calculated as the average difference between the performance in the bootstrap sample and the performance in the original dataset. A lower value indicates less overfitting.  † Calculated by subtracting the optimism value from the apparent performance. This represents the expected performance of the model in future patients from the same population. | | | | |

Note: Analyses were performed using 1,000 bootstrap resamples to assess model stability and overfitting.
